# Functionalized Biomaterials in the Investigation of the Effects of Fluid Shear Forces in the Immune Regulation of Cancer Progression and Metastasis

**DOI:** 10.3390/jfb17020081

**Published:** 2026-02-07

**Authors:** Rayhaneh Afjei, Vassilios I. Sikavitsas

**Affiliations:** 1 School of Chemical, Biological, and Materials Engineering, University of Oklahoma, Norman, OK 73019, USA; rafjei@ou.edu; 2 School of Electrical and Computer Engineering, University of Oklahoma, Norman, OK 73019, USA

**Keywords:** fluid flow, cancer, bioreactor, surface modification

## Abstract

As cancer mortality rates rise globally, malignancies have become the second leading cause of death. Recently, efforts have been made to understand the impact of the tumor microenvironment that involves fluid shear forces. Biomechanical stimulation, which uses shear stress to activate mechanosensitive ion channels, e.g., Piezo1, increases calcium influx into the intracellular space and activates T cells. Novel 3D cancer cultures with T cells have been proposed. Such models use cell/scaffold constructs to recapitulate interactions between cells and the extracellular matrix. In addition, flow perfusion bioreactors investigate the impact of fluid shear forces on immune and/or cancer cells. These bioreactors have biosensors that allow monitoring of immune cell activation. Furthermore, they provide a biomimetic environment for the study of the interaction of T cells and cancer cells. Hence, immune checkpoint inhibitors have demonstrated immunotherapeutic efficacy, but a single-target blockade has often proved insufficient. Co-delivery of CCL19 pDNA and the PD-1/PD-L1 interaction inhibitor BMS-1 using RGD-modified nanocarriers targeting tumor integrins enhanced local antitumor immunity. This review highlights recent insights into how fluid shear stress (FSS) regulates cancer progression and immune responses in three-dimensional in vitro models, with a focus on bioreactors and the surface modification of scaffold materials.

## 1. Introduction

The occurrences of cancer, in addition to its mortality rates, are increasing on a global scale, meaning malignant tumors have become the second most common cause of death, preceding heart disease [1]. Hence, they pose a significant threat to public health. Cancer progresses from a tumor that is localized into a complicated and varied metastasis. This is why such progression is the main reason for the death of cancerous patients [2]. The leading players in the frontline of the war on cancer are T cells, which not only recognize but also destroy tumor cells [3,4]. The development of T cells ignites in the thymus. This is the place where thymocytes undergo a series of selection processes to develop into T cells [5]. In tumor-draining lymph nodes, T follicular helper (Tfh) CD4+ T cells help B cells activate and mature. At the same time, another type of helper CD4+ T cells promote the priming of CD8+ T cells by licensing dendritic cells (DCs) to cross-present antigens [6]. Maintenance of immune homeostasis relies on inhibitory immune checkpoints that regulate the strength and duration of immune signaling and serve as key targets in cancer immunotherapy [7]. In cancer patients, the immune system cannot stop tumor growth, in part due to immune checkpoints, e.g., cytotoxic T lymphocyte-associated protein 4 (CTLA-4) and programmed cell death protein 1 (PD-1) [5].

Various levels of fluid shear stress (FSS) in tissues are experienced by immune cells, rather than in the circulation of blood and lymph. FSS-driven activation of mechanosensitive ion channels elevates intracellular calcium levels, thereby increasing TCR-mediated activation [8]. Mechanical signals within the tumor microenvironment have appeared as regulators of cancer progression and immune function [1]. Recent advances in 3D cultures, engineered biomaterial scaffolds, and flow-perfusion bioreactors enable the observation of these mechanical effects under physiological conditions [9]. By integrating tunable material properties, scaffold architecture, and defined flow regimes, these platforms provide new opportunities to dissect how mechanical and biochemical signals converge to shape cancer-immune interactions across multiple length scales [10].

This review aims to study scaffold-based three-dimensional culture systems and perfusion bioreactors in cancer modeling and to assess how these approaches advance our understanding of metastatic progression and immune regulation, particularly T cell responses [11,12].

### 1.1. T Cell Biology

Hematopoietic stem cells in the bone marrow give rise to common lymphoid progenitors (CLPs), which later differentiate into T cells [13]. Following this, CLPs migrate to the thymus, where they undergo thymopoiesis, including positive and negative selection, ultimately giving rise to mature single-positive naïve CD4+ helper or CD8+ cytotoxic T cells [13,14].

Conventional αβ T cells are characterized as CD4+ helper T cells, mainly considered as helpers for other immune cells, or CD8+ cytotoxic T cells, which destroy their target cells [15]. T cells show an exclusive T cell receptor (TCR) that can recognize small peptide fragments found on major histocompatibility complex (MHC) molecules. CD4+ helper cells are restricted in MHC class II (MHC-II), but CD8+ cytotoxic T cells are restricted in MHC-I [15]. CD8+ T cells are contact-dependent killers, meaning they must be physically inside the tissue of interest to be able to destroy the peptide-MHC class I-bearing target cells [16]. Following migration and extravasation into tissues, T cells are exposed to the local microenvironment, which influences their diversity, fate, and function [16,17]. Infected or malignant cells can be directly eliminated by cytotoxic T lymphocytes, including both CD8+ and CD4+ CTLs, while CD4+ T helper cells primarily orchestrate innate and adaptive immune responses through co-stimulatory signaling and cytokine-mediated communication [18]. For example, upon detection of a severe infection CD4+ and CD8+ T cells differentiate into effector CD8+ T cells that undergo vigorous expansion and get cytotoxic functions. In contrast, those in chronic infection enter an exhaustion state, rapidly losing effector function and developing an elevated inhibitory phenotype [18,19]. Overall, T cell development and function are influenced by biological processes and the surrounding environment that support immune defense.

### 1.2. Checkpoint Regulation

The immune system must be able to differentiate self-cells from non-self-cells to produce an accurate response [20]. Immune responses can be induced by any cell that lacks host-specific recognition patterns [20]. Once a response is given, a healthy immune system will limit its response to prevent opposing events from occurring [20]. Immune checkpoints are regulators of immune responses that act as “brakes,” protecting normal cells from immune attacks while still allowing the immune system to fight infections [21,22]. Cytotoxic T-lymphocyte-associated protein 4 (CTLA-4), programmed cell death protein 1 (PD-1), and programmed death ligand 1 (PD-L1), which can inhibit T cell function when engaged, are some of these checkpoints [21]. The suppressive functions of immune checkpoints are usually dependent on ligand-induced signaling [23]. Two signals, one from the T cell receptor (TCR) and the other from co-stimulatory pathways, activate the T cell immune response [23]. Signals from co-stimulatory pathways amplify the response; hence, the lack of such signals may lead to anergy, meaning the immune cells will be unresponsive to a trigger [20]. The immune response will be reduced and stopped in many ways, including through inhibitory pathways (e.g., the CD28 and PD-1 pathways) [20]. When TCR stimulation is matched by PD-1 engagement, inhibitoryPD-1 signaling interferes with downstream TCR phosphorylation events, leading to rapid signal shutdown and limiting T cell activation [7].

Meanwhile, CTLA-4 is a cell-surface receptor that binds to its ligands, such as B7.1 and B7.2, then limits CD28 signaling, thereby reducing T lymphocyte activity and overpowering the immune response [20]. The first two coinhibitory immune checkpoint proteins that were studied and approved for cancer treatment are PD-1 and CTLA-4 [24]. Other inhibitory checkpoints like Lymphocyte Activation Gene 3 (LAG-3), T Cell Immunoglobulin and Mucin-3 (TIM-3), T Cell Immunoreceptor with Ig and ITIM domains (TIGIT), and V-domain Ig Suppressor of T Cell Activation (VISTA) are still under investigation in order to determine their roles in immune modulation and consider them as potential therapeutic targets [25,26]. Table 1 presents a brief list of immune checkpoints [27]. Immune checkpoints are regulators of T cell activation, while their dysregulation can suppress immune responses.

### 1.3. Fluid Shear Stress

Shear stress is a mechanical factor in both normal physiology and malignancy. Physiological processes, such as blood flow and gut particle movement, generate shear stress that influences the behavior of various tissues [35,36]. The term Piezo is derived from the Greek word “πίεση” (píesi), meaning pressure [37]. Piezo1 and Piezo2 are cation channels that are mechanically activated and form enough homomultimeric complexes to contribute to currents that are mechanically induced [25,38]. Merkel cells (MCs) are specialized epidermal cells that associate with SA1 Aβ low-threshold mechanoreceptors (Aβ-LTMRs), forming Merkel cell–mechanoreceptor complexes [39]. The principal sensor of mechanical forces in mammalian Merkel cells is Piezo2, while Piezo1 mediates mechanically activated cation currents in other cell types, including endothelial cells [40]. The 2021 Nobel Prize in Physiology or Medicine was awarded to Dr. Ardem Patapoutian, who identified Piezo1 as a mechanosensitive ion channel that regulates calcium influx by opening in direct response to membrane tension and cytoskeletal interactions [41]. Piezo1 transduces physical stimuli into biochemical responses due to Calcium influx, given that calcium is a second messenger in signaling pathways [42]. One pathway by which calcium induces apoptosis is mitochondrial dysfunction [43]. Calcium influx activates calpains, which cleave Bcl-2 and convert Bid to tBid, thereby inducing intrinsic apoptosis [43]. Under physiological conditions, Piezo1 mediates endothelial cell responses to shear stress generated by blood flow [44]. Additionally, Piezo1 is involved in mechanosensory processes across the gastrointestinal tract, urinary tract, joints, lungs, and touch perception [45]. Mutations in Piezos that are relevant to severe pathologies in humans also indicate the significance of these ion channels as molecular sensors that are necessary for normal physiological function [46].

**Figure 1:**
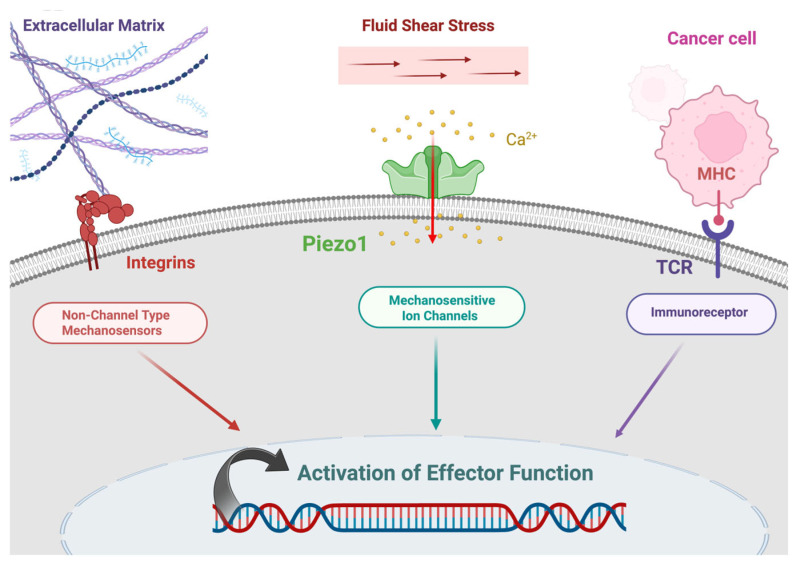
Mechanotransduction in an immune cell. Created in BioRender. Afjei, R. (2026) https://BioRender.com/cndr4cw (accessed on 24 January 2026).

The mechanical properties of integrin were demonstrated in 1993, before which integrin was viewed as a biochemical sensor [47,48]. In 1993, using magnetic beads and magnetic twisting cytometry (MTC) to apply force to the cell membrane, researchers found that cytoskeletal stiffening occurred only with Arg-Gly-Asp (RGD)-coated magnetic beads, implying that cells sense external force through the RGD binding motif. As RGD is a specific recognition site for integrins, this phenomenon suggests that integrins function as mechanosensors. Like integrins, which have both extracellular and intracellular domains, GPCR82 and Piezo1 also function as mechanosensors that mediate outside, in mechanotransduction [49,50]. In summary, fluid shear stress affects the behavior of many cells, including T cells, through mechanosensitive proteins such as Piezo channels and integrins (Figure 1).

## 2. Cancer Metastasis

Metastasis is the main reason for cancer mortality in 90% of cancerous patients, making therapeutic strategies for targeting circulating tumor cells (CTCs) in the bloodstream beneficial [51]. The steps involved in metastasis include invasion, migration, intravasation, dissemination, extravasation, and colonization. Cancer cells circulating in the bloodstream during these processes are named circulating tumor cells (CTCs) [52]. Cancer cells change their motility and degrade the extracellular matrix (ECM) to metastasize from the primary tumor. Proteolytic enzymes, such as metalloproteases, degrade all types of ECM proteins, helping cancer cells exit the tumor and intravasate into the vasculature [53,54].

### 2.1. Fluid Shear Stress and Epithelial to Mesenchymal Transition (EMT)

**Figure 2:**
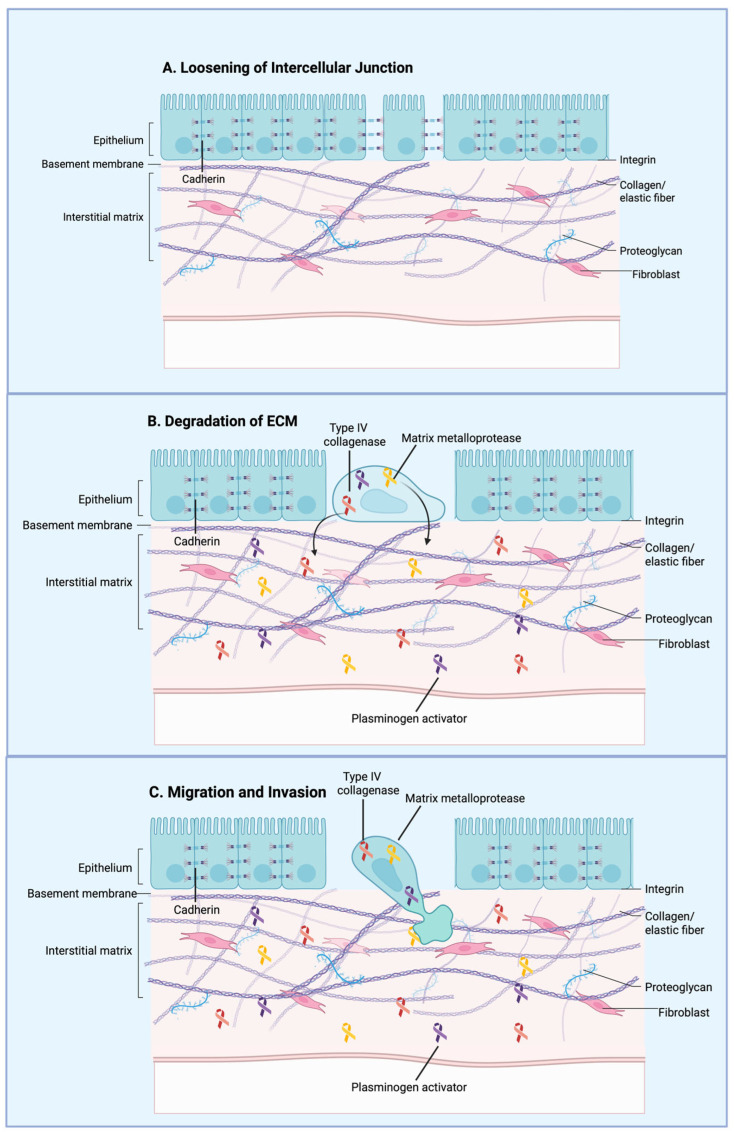
General overview of the epithelial–mesenchymal transition (EMT) during cancer metastasis. Cancer cells lose cell–cell adhesion while acquiring mesenchymal characteristics, including enhanced motility and invasiveness. These mesenchymal-like cells detach from the primary tumor, invade surrounding tissues, intravasate into the bloodstream, and lead to metastatic dissemination. Created in BioRender. Afjei, R. (2026) https://BioRender.com/bpyfnd8 (accessed on 24 January 2026).

Epithelial to Mesenchymal Transition (EMT) promotes cancer cell motility and invasiveness, enabling migration toward the vasculature and intravasation through endothelial junctions [55]. During EMT, epithelial cells experience structural and molecular changes, including basement membrane reorganization, loss of epithelial cell–cell adhesion mediated by E-cadherin, and enhanced expression of mesenchymal proteins such as N-cadherin and vimentin [41]. Increased expression of the transcription factors SNAIL1 and SNAIL2 is associated with elevated phospholipase D2 (PLD2) protein levels, which in turn support cellular migration, survival, and resistance to chemotherapeutic stress [41,56]. Changes in cellular architecture drive upregulation of Piezo1, increasedCa^2+^ influx, and the induction of epithelial-to-mesenchymal transition in breast cancer cells [57]. Methods used to investigate morphology-dependent variations in Ca^2+^ signaling suggest a novel approach for identifying therapeutic agents and targets that can disrupt the interplay between cell shape, signaling pathways, and cellular plasticity [57]. Thus, fluid shear stress plays an important role in EMT by promoting structural changes and migratory behavior in cancer cells (Figure 2).

### 2.2. Fluid Shear Stress and Circulating Tumor Cells (CTCs)

Cancer cells transfer through the bloodstream in the form of tumor cells (CTCs) circulating to a secondary site during metastasis [58]. This movement can be performed as single CTCs or as CTC clusters with cancer-associated fibroblasts (CAFs) [59]. CTCs were first found by Ashworth in 1869 during an autopsy of a metastatic patient. His study concluded that, in contrast to cells of the original tumor, CTCs are found far from the tumor [60]. While in circulation, circulating tumor cells (CTCs) are exposed to fluid shear stress (FSS) that exceeds 1000 dyn/cm^2^ (~100 Pa) [8]. These levels are generally lethal to most CTCs [7], but a subpopulation begins to resist these mechanical forces, leading to survival and later colonization of distant organs [11].

Cancer cells are exposed to highly adjustable shear stresses across vascular environments, in a range between 4–12 dyne/cm^2^ in lymphatic vessels and lower than 0.1 dyne/cm^2^ in solid tumors, to 0.5–30 dyne/cm^2^ in the circulation, with temporary peaks going over 1000 dyne/cm^2^ at main junctions and inside the heart [56]. CTCs encounter high fluid shear stress (FSS) in the bloodstream, which eliminates most cells, leaving a survival rate of less than 0.01% to colonize secondary tissues [56]. CTCs’ liquid biopsy and circulating tumor DNA (ctDNA) are noninvasive and provide information about the tumor [61]. Mutations in genes such as KRAS, EGFR, and HER2, as well as in the estrogen receptor (ER) genes, can be identified via CTC screening [61].

Novel therapeutic approaches have been proposed that selectively drop CTCs to limit hematogenous metastasis [9]. Membrane injury may be induced by drug-based strategies, which increase CTC deformability, thereby reducing CTC survival under shear stress [9].

### 2.3. In Vitro Models That Approximate Physiological Conditions in Cancer Metastasis

Two-dimensional (2D) cell culture platforms are widely used due to their experimental simplicity, cost-effectiveness, and applicability across multiple cell types. Although these platforms provide a fast and simple cell-expansion method, they lack several vital mechanisms of natural tissue anatomy [61]. For example, the absence of extracellular matrix (ECM) support in 2D cultures induces substantial alterations in cellular structure and downstream gene regulatory activity [62].

Recognizing the limitations of two-dimensional cultures, three-dimensional (3D) in vitro models have been established as platforms that more closely resemble in vivo systems. In this context, spheroids derived from patient-derived tumor cells have been used for treatment selection and screening in various cancers [63,64]. These platforms are designed to approximate physiological cellular environments by incorporating extracellular matrix (ECM) components [61]. As a result, transcriptional activity and structural organization differ between 2D and 3D cultures. In 2D cultures, cells have uniform access to oxygen and nutrients due to monolayer growth [62,65].

In contrast, 3D cultures—particularly avascular models such as tumor spheroids—exhibit diffusion limitations that restrict oxygen and nutrient availability, thereby limiting cellular proliferation [66]. Insufficient oxygen penetration in these 3D cultures leads to metabolic and genetic adaptations within the hypoxic core [67]. Additionally, 3D cultures allow the integration of extracellular matrix (ECM) components, which regulate the expression of adhesion-related genes, including E-cadherin [68]. These conditions also facilitate epithelial-to-mesenchymal transition (EMT) in cancer cells, a process more commonly observed in 3D systems than in 2D cultures [52]. EMT has been strongly associated with tumor progression, invasive behavior, metastatic potential, and resistance to therapeutic interventions [69]. Several important considerations arise when comparing 3D cultures with animal models, the most important being the fact that nude mice are immunodeficient and therefore lack a functional immune system. Tumors injected into nude mice have been widely applied for anticancer drug evaluation and cytotoxic drug screening [70].

In many cases, such models provide limited insight into immune-mediated therapeutic responses, highlighting a key distinction between animal models and 3D in vitro platforms [71]. This has shown the limitations of the nude mouse model and highlighted the need for novel, advanced alternative platforms [72]. This gap has been addressed through the development of 3D in vitro models that enable more precise cancer research [73]. Consequently, 3D in vitro tumor models provide physiologically relevant tools in cancer biology that can be used that hold great promise in screening cancer treatments as we moved toward personalized medicine.

## 3. Perfusion Bioreactor for Studying T Cell Responses to FSS

Bioreactors provide culture environments that support a wide range of cell types and experimental conditions, making them suitable for laboratory research. Their design allows for the integration of various components, including sensors, to meet specific experimental requirements. The ability to incorporate multiple sensing modalities enhances process control and helps with continuous monitoring [74]. In addition, integrated sensors generate critical datasets that improve measurement accuracy and allow near-real-time assessment of culture conditions [75]. Flow-perfusion bioreactors have been widely used in tissue engineering studies, and they have been adopted in the culture of three-dimensional cancer models. They are straightforward to run and can expose cells to controlled fluid shear stress (FSS). Such mechanical stimulation is essential for the successful culture of many cell types, particularly in osteogenic applications, where shear forces promote bone formation and enhance mesenchymal stem cell (MSC) differentiation toward osteoblasts [76]. Flow-perfusion bioreactors show fluid-flow properties that overcome most diffusional mass-transport limitations in conventional 3D in vitro scaffold designs [76,77].

**Figure 3:**
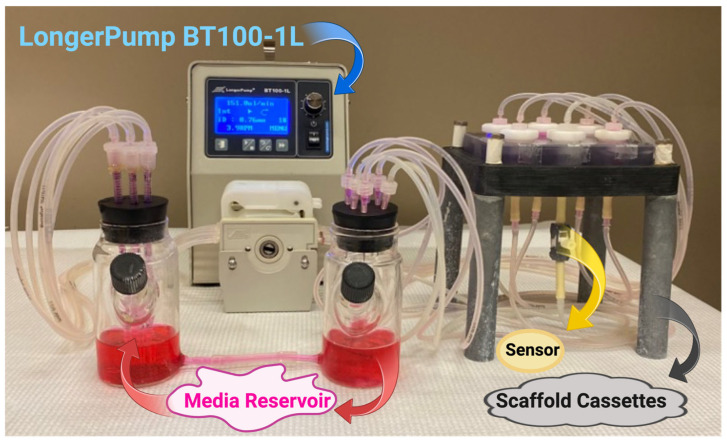
Flow perfusion bioreactor. Created in BioRender. Afjei, R. (2026). https://BioRender.com/nbmkxsc (accessed on 24 January 2026).

Flow-perfusion bioreactors offer distinct experimental platforms compared with conventional 2D in vitro systems and microfluidic devices (Table 2) [78]. Although microfluidic platforms, such as organ-on-a-chip models, have shown efficacy in cancer diagnostics and therapeutic screening [78,79], they often lack the scale and structural complexity needed to fully recapitulate both micro- and macro-level tumor environments. In contrast, flow-perfusion bioreactors keep the key advantages of microfluidic systems while running at the macroscale, enabling a more comprehensive representation of the tumor microenvironment across multiple length scales [80,81]. Additionally, flow-perfusion configurations allow the integration of many interconnected scaffolds and the establishment of variable upstream conditions (Figure 3). This modularity enables investigation of cell–cell signaling within a single bioreactor via controlled fluid flow. For instance, cancer cells may be positioned upstream of immune cells, allowing intercellular signaling to be assessed via bulk media sampling. Compared with static 3D cultures (Table 2), flow-perfusion systems have been shown to enhance cell proliferation and cellular homogeneity within tissue-like constructs, while promoting morphologies and phenotypes that more closely resemble those seen in xenograft models [82].

Furthermore, biomechanical stimulation induced by shear stress is often ignored as a contributor to tumor progression [77,78], which 3D macroscale systems provide a controlled in vitro environment for studying these dynamic mechanical cues. T cell responses to fluid shear stress (FSS) mainly involve integrin-mediated adhesion and cell motility. Levels of FSS exceeding 1 dyne/cm^2^ are needed to support VLA-4/VCAM-1– and LFA-1/ICAM-1–dependent T cell attachment and rolling along the endothelium [87]. These findings underscore the critical role of FSS in regulating T cell immune surveillance, migration, and certain arrests at inflammatory sites [87]. Mechanosensitive ion channels, including Piezo1, send fluid shear stress into intracellular biochemical signaling pathways, thereby modulating key T cell functions such as enhanced activation, proliferation and differentiation [88]. The T cell receptor (TCR) and B cell receptor (BCR) are immunoreceptors that differentiate between antigens with high specificity and begin the activation of T and B cells. In addition, given that these receptors are mechanotransducers, their function is dependent upon reactions provided in responses to mechanical stimuli [89].

There is evidentiary support that signifies the fact that FSS surges T cell receptor (TCR) signaling via boosted activation of the NF-κB, AP-1, and NFAT pathways [88]. A large amount of research has shown how sensitive blood cells, such as s and platelets, are to shear stress, in cases where proliferation and/or cytokine release have risen [90]. A potential pathophysiological link between T cell activation and fluid shear stress (FSS) is suggested by the association among hypertension, disturbed blood flow, and autoimmune diseases [42]. T cell activation was found to be regulated by calcium via the mechanosensitive ion channel Piezo1. This shows that Piezo1 and related channels can serve as therapeutic targets in autoimmune disease and in adoptive T cell therapy [42]. Following exposure to fluid shear stress (FSS), IFN-γ and IL-2 levels are elevated. IFN-γ promotes apoptosis in cancer cells, while IL-2 supports the sustained activity of the chimeric antigen receptor (CAR) T cells after reinfusion [42]. With FSS being an important regulator of T cell behavior, the development of flow perfusion bioreactors that can generate the appropriate fluid dynamic environment becomes an important player in the study of 3D tumor models.

## 4. T Cells in the Tumor Microenvironment

Tumor cells drive substantial molecular, cellular, and physical changes within host tissues during the early stages of tumor development, and a dynamic and reciprocal interplay appears among cancer cells and components of the tumor microenvironment (TME), promoting tumor cell survival, local invasion, and metastatic spread [91]. The TME is highly complicated and constantly developing, surrounding not only stromal, fibroblastic and endothelial compartments, but also cells of both the innate and adaptive immune systems [92].

### 4.1. Cancer Cell–T Cell Direct Interaction

“Hot tumors” are defined by extensive lymphocyte infiltration within the tumor microenvironment (TME), while “cold tumors” show minimal immune cell infiltration and are considered immunologically ignorant [93]. Hot tumors are particularly enriched in T cells, with substantial intratumoral accumulation and robust antitumor immune activity [93]. T lymphocytes, including both CD4+ and CD8+ subsets, create a critical part of the TME [94]. However, in established tumors, CD8+ T cells often become a dysfunctional or “exhausted” phenotype. T cell exhaustion is characterized by upregulation of multiple immune checkpoint receptors, such as PD-1, LAG-3, and TIM-3, along with impaired proliferation and diminished production of effector cytokines, including IFN-γ [95].

The high metabolic demands of rapidly growing tumor cells reduce the availability of essential nutrients within the TME, thereby limiting glucose for activated T cells needed to support proliferation and effector function [96]. Moreover, the abnormal tumor vasculature leads to heterogeneous nutrient distribution, further impairing effector T cell metabolism. Nutrient deprivation within the TME, combined with chronic antigenic stimulation, drives metabolic reprogramming of effector T cells, resulting in reduced glucose uptake, elevated reactive oxygen species levels, loss of functional ability, loss of mitochondrial mass, and shortened effector function in T cells [90,93]. Thus, direct interactions between cancer cells and T cells within the tumor microenvironment shape immune activity and often lead to T cell dysfunction.

### 4.2. Cancer Cell–T Cell Interaction via Cancer Exosome

Exosomes are nanoscale extracellular vesicles, typically ranging from 30 to 160 nm in diameter, that are released by most cell types and enclosed by a phospholipid bilayer [97]. These vesicles carry diverse molecular cargo, including DNA, small RNAs, proteins, and other bioactive molecules, enabling intercellular communication through the transfer of nucleic acids and proteins. In cancer, exosomes contribute to tumor progression and metastatic dissemination by delivering tumor-associated molecular signals to recipient cells [94]. Tumor-derived exosomes (TEXs), which originate from malignant cells, have been shown to enhance tumor growth by interacting with both cancerous and non-malignant cells throughout the body, thereby influencing systemic processes involved in cancer progression. Cancer-derived exosomes have been reported to facilitate metastasis through multiple mechanisms, including modulation of the immune system, promotion of epithelial-to-mesenchymal transition (EMT), organotropism, and angiogenesis [98]. Tumor-derived exosomes can decrease cytotoxic CD8+ T cell populations and promote the conversion of CD4+ helper T cells into immunosuppressive regulatory T cells (Tregs), thereby enhancing the tumor microenvironment’s ability for immune evasion. Exosomes have also been shown to inhibit T cell activation, primarily through targeting TGF-β signaling.

Additionally, exosomes released from metastatic melanomas can express surface PD-L1, contributing to tumor progression by suppressing CD8+ T cell function [99]. Tumor-derived exosomes have cancer-associated antigens that can trigger immune responses. Several studies [100,101] have suggested that these exosomes may indirectly interact with CD8+ T cells via antigen-presenting cells (APCs) through processes such as cross-presentation or cross-dressing [102,103]. This T cell deactivation is associated with reduced production of effector cytokines, including IL-2 and TNF, or with increased levels of IL-6 [104,105]. Cancer exosomes are often neglected components that affect T cell activity in favor of immune evasion and tumor growth.

### 4.3. In Vitro Models of Cancer Cell–T Cell Interaction

**Figure 4:**
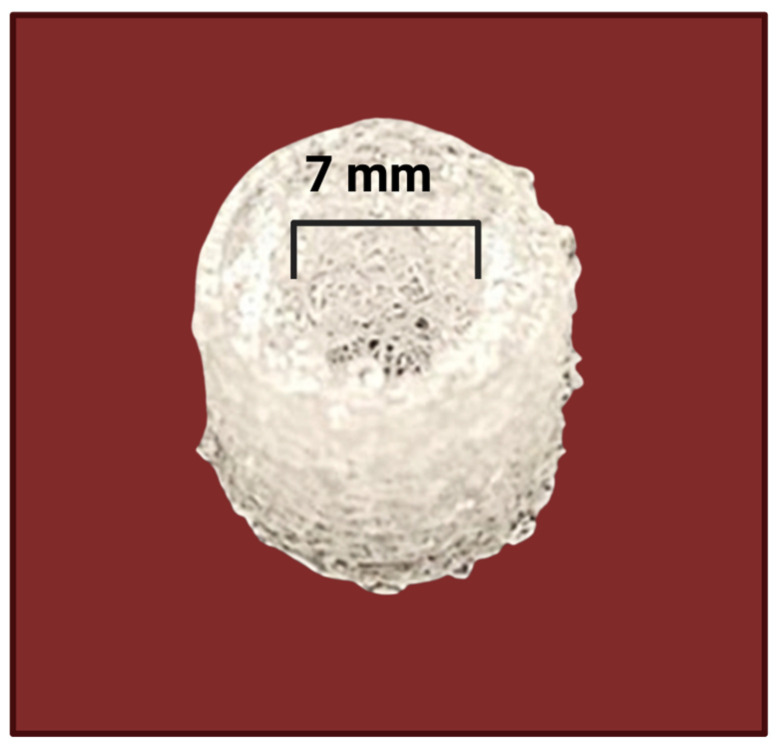
3D-printed PLLA mesh. Created in BioRender. Afjei, R. (2026) https://BioRender.com/t3prfq3 (accessed on 24 January 2026).

Scaffolds that can support perfusion have become valuable tools for promoting cell adhesion and for recreating key features of the tumor microenvironment. Materials such as hydrogels [106], synthetic rigid scaffolds, including polycaprolactone (PCL) and poly (lactic-co-glycolic acid) (PLGA) (Figure 4) [107], and modified hydrogel systems [108] have all been successfully used to culture specific cancer cell types. More importantly, scaffold characteristics—including stiffness, pore size, fiber dimensions, and interconnectivity—play a critical role in shaping cellular behavior and culture outcomes [109]. One solution to this problem has been the use of 3D printing, which would help accurately build one-shaped, or possibly unsystematic, scaffolds that attempt to mimic microenvironments. Even though some of the material in the 3D printing scaffold may have restrictions in adhesion or microenvironment, the research has been successful in illustrating the feasibility of changes that can be made to the surface of specific complex polymers, such as poly-(lactic acid) (PLA) and poly-(ε-caprolactone) (PCL) using peptides and other linkers [110]. Scaffold models provide physiologically relevant platforms for studying cancer cell–T cell interactions within the tumor microenvironment.

Scientists have developed a novel experimental approach to examine how T cells respond to cancer-derived exosomes. This strategy uses 3D-printed, RGD-functionalized PLLA scaffolds integrated into a flow-perfusion bioreactor system. Using this platform, investigators determined the critical exosome concentration required to suppress IL-2 production by utilizing activated CD8+ T cells under both static and dynamic flow conditions (Table 3) [111].

## 5. Reinvigoration of Suppressed T Cells

Many human cancer types, especially those with higher mutational burden, can be attacked against by CD8+ T cells. This means that T cell infiltrations that previously existed can serve as a positive prognostic indicator across a variety of cancers. Furthermore, PD-L1 expression in tumors and T cell responses are associated with each other, where CD8+ T cell responses cannot be eliminated by cancer cells, making CD8+ T cells dysfunctional or exhausted [112].

Exhausted CD8+ T cells keep effector ability but are characterized by a differentiation path that differs from effector and memory T cells. Rather than owning effector or memory functions, they show dysfunctional cytokine production and limited cytotoxic potential. This phenotype is associated with sustained expression of inhibitory receptors, including programmed cell death protein 1 (PD-1), which constrain T cell signaling and function [112]. Chronic antigen exposure within the tumor microenvironment leads to the differentiation of exhausted CD8+ T cells, with reduced proliferative ability and longevity, thereby losing durable antitumor immunity [113].

### 5.1. Mechanotransducive Modulation of T Cell Activity

Fluid shear stress (FSS) can significantly enhance primary T cell activation via the mechanosensitive ion channel Piezo1. This will lead to increased calcium signaling and downstream activation of key transcription factors over 10 days [88].

Integrins function as essential mechanosensors that help the cell–extracellular matrix (ECM) adhesion by binding ECM components and transducing mechanical cues linked to ECM rigidity and composition into intracellular signals. In T cells, the integrin LFA-1 (lymphocyte function-associated antigen-1) is essential for adhesion to antigen-presenting cells (APCs) and contributes to the formation and stabilization of the immunological synapse. Mechanical forces generated during T cell receptor (TCR) engagement increase LFA-1 affinity for its ligand, ICAM-1, thereby promoting T cell activation, adhesion, and migration at inflamed sites.

Consequently, advanced biomimetic hydrogels functionalized with integrin-binding peptides have been developed to improve T cell activation and proliferation in vivo, highlighting their prospective use for cellular therapies in the future [88]. In addition, ex vivo T cell activation remains a critical step in producing adoptive T cell immunotherapies. Short, one-hour fluid shear stress (FSS) exposures, when combined with soluble and bead-bound CD3/CD28 stimulation, amplify key signaling protein activation and elevate the expression of cytokines essential for sustained T cell function [43]. As a result, fluid shear stress regulates T cell activation through mechanotransduction mechanisms.

### 5.2. Immune Checkpoint-Mediated Reversal of T Cell Exhaustion

Prolonged exposure to tumor-derived antigens can drive CD8+ T cell exhaustion, characterized by elevated expression of inhibitory receptors, such as PD-1, and a consequent reduction in antitumor efficacy. Blocking the PD-1–PD-L1 axis can restore function in exhausted CD8+ T cells (TEXs), thereby improving effector activity and enhancing tumor control [114]. Although PD-1–PD-L1 signaling is a central axis limiting T cell activity, a broader network of coinhibitory receptor–ligand interactions contribute to dampened antitumor responses across the tumor microenvironment, acting directly or indirectly. Among these receptors, TIM-3, LAG-3, CTLA-4, and TIGIT have ligands expressed on malignant cells or immune cells in diverse tumors, creating an inhibitory environment that modulates T cell function at different stages of activation and may underlie resistance to the PD-1/PD-L1 blockade [115]. In particular, TIGIT is often seen on dysfunctional CD8+ T cells in human tumors, and its principal ligand, CD155, is overexpressed in multiple cancer types and impairs immune surveillance through TIGIT engagement. Preclinical data suggest that a TIGIT blockade can augment CD8+ T cell responses, though inhibition of TIGIT alone often does not restore the tumor fully infiltrating CD8+ T cell function. Importantly, combining TIGIT inhibitors with a PD-1/PD-L1 blockade produces synergistic enhancement of antitumor CD8+ T cell immunity, including in models resistant to PD-1 blockade [116]. Immune checkpoint inhibitors primarily disrupt the exhaustion program but do not directly address ongoing chronic antigen stimulation, metabolic disorders, or genetic drivers of exhaustion; as a result, persistent antigen exposure can sustain surface inhibitory receptor expression and limit durable reversal of exhaustion [117]. In turn, targeting multiple inhibitory receptors offers an effective strategy for reversing T cell exhaustion.

### 5.3. Genetic Engineering to Add Receptors, as in CAR T Cell Therapy

The adenovirus wild-type fiber was engineered to include an Arg-Gly-Asp (RGD) motif within the HI loop of the fiber knob domain, enabling recognition by the αvβ3 and αvβ5 integrins during cell entry. T cells isolated from mouse splenocytes and human peripheral blood mononuclear cells (PBMCs) were efficiently transduced by Ad-RGD viruses compared with adenoviruses carrying wild-type fibers. Oncolytic Ad-RGD viruses also replicated within transduced T cells, as evidenced by increased E1A gene copies, higher hexon protein expression, viral progeny production, and enhanced cytotoxicity. In vivo, treatment of mice with Ad-RGD resulted in greater infection of splenic T cells than wild-type fiber viruses. When combined, these findings indicate that Ad-RGD adenoviruses can efficiently transduce T cells and utilize their replicative machinery [116]. Taken together, CAR T cells provide targeted cancer therapies enhancing T cell function with impressive clinical outcomes mainly in myelomas and lymphomas.

## 6. Therapeutic Perspectives

Tumor cells often express cell-surface immunosuppressive checkpoint molecules to evade immune detection, leading to downregulated antitumor immune responses [13]. Consequently, immune checkpoint inhibitors have shown potential as immunotherapeutic agents across diverse cancers [15]. Although these inhibitors can yield clinical benefits, a single-agent blockade often proves insufficient due to rapid resistance development [16]. This highlights the need to optimize combination strategies that target multiple immune regulatory pathways to overcome immune-related adverse events, reprogram the tumor microenvironment (TME) toward a pro-immune state, and elicit durable antitumor immunity. In this context, one study developed a targeted immunogene therapy that combines an immunostimulatory chemokine CCL19-encoding plasmid DNA (CCL19 pDNA) with BMS-1, a small-molecule inhibitor of the PD-1/PD-L1 interaction, to activate local immune responses and control the immunosuppressive TME. They designed nanocarriers engineered to present Cyclo (Arg-Gly-Asp-d-Phe-Lys) (cRDGfk, i.e., RGD) peptides that specifically bind to αvβ3 integrin, which is highly expressed on tumor cells and tumor vasculature [21]. Tumor-targeting nanoparticle design carrying CCL19 pDNA and BMS-1 is directed to promote a localized anticancer immune response. This nanoscale immunogene therapy provides several benefits. First, tumor-targeting RGD designs enhance nanoparticle accumulation at tumor sites and improve intracellular delivery efficiency of CCL19 pDNA and BMS-1, enabling tumor cells to produce biologically active CCL19. Second, administration of CCL19 pDNA and the consequent activation of immune cells increases IFN-γ secretion, which has been shown to induce PD-L1 expression on tumor cells. This reciprocal upregulation can, in turn, dampen local T cell activation and attenuate local antitumor immunity [117]. These dynamics highlight the importance of carefully coordinating a checkpoint blockade with immunogene delivery to maximize therapeutic efficacy [118,119].

## 7. Future Directions

Despite the numerous advancements in the use of bioreactors and biopolymers in developing 3D in vitro tumor models, significant hurdles remain to move toward their use in personalized oncology treatments. Regulatory agencies need to be convinced that patient tumor biopsies cultured as 3D spheroids within scaffold-supported bioreactors to screen different chemotherapeutic drugs, combinations, and dosages can assist in identifying the most effective treatment for individual patients. Collaborative clinical studies between biomaterials experts, bioengineers, biologists, and oncologists will be needed to clearly demonstrate the ability of these models to predict optimal cancer treatments [120]. Cancer mechanotherapy is a new field of medicine that uses mechanical stimuli, such as fluid shear stress (FSS) and ultrasound, to activate immune cells and sensitize cancer cells to chemotherapy [8]. Such approaches are still at preclinical stages and require further validation in multistage clinical trials to become available to patients. CAR-T cell therapies revolutionized cancer treatments but are still not effective in many types of cancer, especially solid tumors, mainly because the fibrotic tumor microenvironment (TME) blocks immune cell infiltration. Recent studies show that biomechanical cues such as fluid shear stress (FSS) can activate immune cells. Using FSS-based platforms like perfusion bioreactors and modifying the TME in 3D cultures may optimize the function of CAR-T cells and their tumor penetration [121].

## Figures and Tables

**Table 1:** Immune checkpoint receptors and their ligand-expressing cells.

Receptor	Expressing Cells	Ligands	Ligand-Expressing Cells	References
PD1	T cells, B cells	PD-L1, PD-L2	APC, Tumor cells	[22,28]
CTLA4	T cells	CD80, CD86	APC	[29]
LAG-3	T cells, NK cells	MHC class II	APC	[30]
TIM-3	T cells, DC, NK	Galectin-9	Stromal cells, Tumor cells	[31,32]
TIGIT	T cells, NK cells	CD155, CD122	APC, T cells	[33,34]

**Table 2:** Comparison of in vitro platforms for modeling cancer metastasis.

Model	Key Features	Strengths	Limitations	References
2D Culture	Monolayer cell growth	Low-cost, rapid cell expansion	Poor physiological relevance	[83,84]
3D Culture	Cell growth in ECM	Tumor architecture, hypoxia	Static conditions	[83,84]
Microfluidics Systems	Microscale channels	Real-time monitoring	Simplified tissue structure	[85]
Perfusion Bioreactors	3D scaffolds with controlled flow	Mechanical stimulation	More complex setup, higher cost	[86]

**Table 3:** Scaffold platforms used in perfusion-compatible tumor and immune models.

Scaffold	Material	Culture Mode	References
Hydrogel scaffolds	Collagen-based hydrogel	Static/perfusion	[106]
Synthetic polymer scaffold	PCL, PLGA	Perfusion	[107]
3D-printed polymer scaffold	PLA	Static/perfusion	[110]
Peptide-functionalized scaffold	RGD-modified PLLA	Perfusion	[111]

## Data Availability

Data sharing not applicable.
